# Development of a Memory Clinic at an Aboriginal Community‐Controlled Health Service: Profile of the First Patients

**DOI:** 10.1111/ajag.70106

**Published:** 2025-11-02

**Authors:** Zoë Hyde, Leon Flicker, Richelle Douglas, Sadia Rind, Nadia Rind, Dina LoGiudice, Kate Smith

**Affiliations:** ^1^ Centre for Aboriginal Medical and Dental Health, Medical School University of Western Australia Perth Western Australia Australia; ^2^ Western Australian Centre for Health and Ageing, Medical School University of Western Australia Perth Western Australia Australia; ^3^ Derbarl Yerrigan Health Service Perth Western Australia Australia; ^4^ Department of Medicine ‐ Royal Melbourne Hospital University of Melbourne Melbourne Victoria Australia

**Keywords:** aging, Alzheimer disease, Australian Aboriginal and Torres Strait Islander peoples, dementia, indigenous peoples

## Abstract

**Objectives:**

Dementia is the leading cause of burden of disease in older Australians. Older Aboriginal and Torres Strait Islander people experience an increased risk of cognitive impairment and dementia. This article describes the clinical profile of the first patients seen at a memory clinic established in an Aboriginal community‐controlled health service (ACCHS) in metropolitan Perth, Western Australia.

**Methods:**

This was an audit of 64 patients attending a memory clinic between March 2020 and February 2023 (inclusive).

**Results:**

The median age of patients was 67.7 years (range 35–95 years; interquartile range [IQR] 13.4 years) and 34 (53%) were female. The majority (94%) were living independently. Thirty‐four patients (53%; 95% confidence interval 41%–65%) were diagnosed with cognitive impairment. A further six (9%) were diagnosed with depression without cognitive impairment. The most common diagnoses in cognitively impaired patients were cognitive impairment not dementia (CIND; 27%); mild neurocognitive disorder (21%); dementia due to Alzheimer's disease (15%); Alzheimer's disease dementia, mixed type (9%); and other mixed dementias (9%). Women were slightly more likely than men to have cognitive impairment (56% vs. 52%), although this was not statistically significant (*p* = 0.74). The number of Aboriginal people seen in the clinic's first 3 years of operation was over 12 times that seen at a nearby hospital‐based service during the same period.

**Conclusions:**

A memory clinic located within an ACCHS was well‐attended and fulfilled a need not met by mainstream services. The successful model described in this article could be adopted by other Aboriginal health services.

**Summary**

- Practice impact
  ○ Aboriginal and Torres Strait Islander people with concerns about their cognition may experience challenges accessing appropriate health care.
  ○ However, Aboriginal community‐controlled health services can provide accessible and culturally safe care.
  ○ This clinical audit has demonstrated an embedded memory clinic can provide timely, comprehensive assessment and care to an underserved population.

## 1 Introduction

Dementia is the leading cause of burden of disease for Australians aged 65 years and older [1]. Age‐ and sex‐standardized estimates of dementia prevalence suggest that between 21.4 and 65.9 people per 1000 Australians are currently living with dementia [2]. Markedly higher dementia prevalence and incidence (three to five times that of other Australians), and a younger age of onset, have been consistently documented for Aboriginal and Torres Strait Islander peoples [3, 4, 5, 6, 7]. Greater exposure to risk factors, including hypertension, diabetes, head injury, ear disease and hearing impairment, socioeconomic disadvantage, and intergenerational trauma, including the effects of colonization and policies aimed at the removal of children from their families, likely underlies this disparity [8, 9].

Memory clinics are effective multidisciplinary services providing comprehensive assessment of dementia and cognitive impairment, timely diagnosis, and management of care [10]. Despite increased rates of dementia, Aboriginal and Torres Strait Islander peoples are less likely to access government‐funded dementia and aged care services and are half as likely to access specialist medical care compared their non‐Indigenous counterparts [11, 12]. Barriers to care include a lack of access to culturally safe services, fear and stigma, poor health service dementia literacy, mistrust of mainstream services, and financial considerations [13].

Aboriginal community‐controlled health services (ACCHSs) provide best practice, holistic, and culturally appropriate primary health care to Aboriginal and Torres Strait Islander communities [14]. Aboriginal community‐controlled health services have a well‐documented impact on reducing barriers to care and can also enhance the effectiveness of the wider health system by delivering programs in partnership with mainstream health services [14]. Author LF co‐leads the only tertiary memory clinic in the state at Royal Perth Hospital (RPH). Over 2 years throughout 2018 and 2019, only three Aboriginal patients were reviewed at the RPH clinic. In an effort to address this service gap, an ACCHS partnered with authors LF and KS to establish a memory clinic in a central metropolitan location. This paper describes the development of the service and the profile of the first patients seen.

## 2 Methods

### 2.1 Consultation Phase

The need for a specialist service for Aboriginal and Torres Strait Islander people living in Perth with possible dementia was identified in a Theory of Change service provider workshop held in November 2018. This workshop was attended by six staff members from Derbarl Yerrigan Health Service (Derbarl), which is an ACCHS in metropolitan Perth, and four University of Western Australia researchers. The focus of the workshop was the co‐design of a Theory of Change framework for a dementia risk management and prevention program for Aboriginal Australians (DAMPAA), to be based at Derbarl [15]. This workshop identified the resources, activities and assumptions required for the program to have the desired outcomes and impact on reducing dementia rates. The DAMPAA program was open to Aboriginal and Torres Strait Islander people with no major cognitive impairment, as assessed by the Kimberley Indigenous Cognitive Assessment (KICA) tool [16, 17], and where required, a geriatrician review to exclude potential participants with dementia. Key facilitators identified by Derbarl staff members to enable the success of the program were culturally safe referral pathways and specialist care [15]. This included clear referral pathways from Derbarl into the DAMPAA program and vice versa, with DAMPAA Aboriginal Health Practitioners initially based at the Derbarl East Perth clinic, and provision of culturally safe geriatrician reviews and care for people screened as potentially having dementia, or who might develop dementia during the DAMPAA program. Elements that supported culturally safe care included the geriatrician reviews being conducted in a community‐controlled health service, involvement of Aboriginal Health Practitioners, use of culturally informed and validated cognitive tools, and clinic staff being skilled in applying trauma‐informed methods of connecting and communicating with Elders and their families.

In December 2019, the lead DAMPAA investigator (KS) commenced discussions regarding starting a regular Derbarl‐based geriatrician clinic with the new Derbarl medical director (RD) and co‐investigator and study geriatrician LF. The clinic was supported by Derbarl's Chief Executive Officer, Derbarl's Board of Directors, and the Head of the Department of Geriatric Medicine at Royal Perth Hospital. In February 2020, all parties agreed to commence a monthly memory clinic at Derbarl.

### 2.2 Implementation

A memory clinic was established in February 2020 at the East Perth site of Derbarl. A Derbarl Aboriginal Health Practitioner (AHP) was employed to work at the clinic, to contact patients and organize transport through Derbarl, and to complete a KICA assessment prior to the patient attending the clinic. The first patient attended the clinic in March 2020.

### 2.3 Clinic Overview

The clinic ran on a monthly basis for one afternoon session. The reception area at the clinic site is culturally appropriate for Aboriginal clients, with Aboriginal staff members and artwork. Referrals to the clinic were accepted from the treating general practitioners at any of the four clinical sites of Derbarl. Preliminary assessment was made by an AHP (SR and NR). A geriatrician then conducted further cognitive assessment, as appropriate, with the Mini‐Mental State Examination (MMSE) [18] and/or KICA [16, 17]. The service initially included the agreed participation of a dementia specialist nurse, but this person was withdrawn after an initial 3 months because of the tertiary hospital's funding constraints.

### 2.4 Ethical Approval

This work was conducted as part of a clinical audit process; informed consent was not required from participants. Approval was granted by the Comprehensive Care Committee, Royal Perth Bentley Group, East Metropolitan Health Service (approval number: 50416). Reporting of the clinical audit was authorized by Derbarl Yerrigan Health Service's Board of Directors and Chief Executive Officer.

### 2.5 Statistical Analysis

Medical records of patients accessing the service from its inception to February 2023 (inclusive) were reviewed by LF and coded by KS and ZH. The International Classification of Diseases, Eleventh Revision (ICD‐11), was used for diagnostic criteria [19]. Data were then analyzed using Stata, version 17.0 (StataCorp, College Station, Texas). Summary statistics are presented as number and percentage for categorical variables and median and interquartile range (IQR) for continuous variables. Differences in categorical variables between groups were assessed with Pearson's chi‐squared test, whereas the Mann–Whitney *U*‐test was used for continuous variables. Statistical significance was set at 5%.

## 3 Results

A total of 65 people attended the clinic during its first 3 years of operation. One person was the non‐Aboriginal partner of an Aboriginal Derbarl client, and as such was excluded from further analysis. The sociodemographic and clinical characteristics of the remaining 64 people are shown in Table 1. Their median age was 67.7 years (range 35–95 years; IQR 13.4 years) and 34 (53%) were female. Women were slightly older than men, but this was not statistically significant (median 69.0 [IQR 19.3] vs. 67.2 [IQR 10.7] years; *p* = 0.58). The majority of patients lived independently (94%) and most were seen face‐to‐face at the clinic. Three people (5%) were assessed via telephone interview owing to the coronavirus disease 2019 (COVID‐19) pandemic. During history taking, it emerged that at least 21 people (33%) were members of the Stolen Generations (This question was not asked routinely by the geriatrician, due to the distress that this may cause clients). Risk factors for cognitive impairment were common, particularly diabetes, which affected 42% of individuals.

**TABLE 1:** Demographic and clinical characteristics of people attending the clinic.

Characteristic	*n* (%)
Age (years)	
≤ 49	5 (8)
50–59	11 (17)
60–69	21 (33)
70–79	21 (33)
≥ 80	6 (9)
Sex	
Male	30 (47)
Female	34 (53)
Diagnosis	
No impairment	23 (36)
CIND	9 (14)
Mild neurocognitive disorder	7 (11)
Depression	6 (9)
Mixed dementia^a^	6 (9)
Dementia due to Alzheimer's disease	5 (8)
Disorders of intellectual development	3 (5)
Dementia due to use of alcohol	2 (3)
Dementia due to cerebrovascular disease	1 (2)
Other specified neurocognitive disorders	1 (2)
Refused assessment	1 (2)
Cognitive impairment	34 (53)
Living situation	
Community	60 (94)
Homeless	2 (3)
Hostel	2 (3)
Stolen generation	21 (33)
Hypertension	5 (8)
Heart problem(s)	11 (17)
Stroke	11 (17)
Diabetes	27 (42)
Urinary incontinence	2 (3)
Fecal incontinence	1 (2)
Renal failure	2 (3)
Depression	15 (23)
Current or past smoker	16 (25)
Current or past use of alcohol	26 (41)
Requires assistance with activities of daily living	7 (11)
Referral made	28 (44)

*Note:* Data were collected through history taking and review of medical records.

Abbreviation: CIND, cognitive impairment not dementia.

^a^ Mixed dementias comprised Alzheimer's disease dementia, mixed type, with other nonvascular etiologies (6D80.3), and dementia due to use of alcohol (6D84.0); Alzheimer's disease dementia, mixed type, with cerebrovascular disease (6D80.2); Alzheimer's disease dementia, mixed type, with other nonvascular etiologies (6D80.3), dementia due to use of alcohol (6D84.0), and other specified neurocognitive disorders (6E0Y); dementia due to use of alcohol (6D84.0), and dementia due to cerebrovascular disease (6D81); dementia due to use of alcohol (6D84.0), and other specified neurocognitive disorders (6E0Y); and dementia due to use of alcohol (6D84.0), and disorders of intellectual development, unspecified (6A00.Z).

**FIGURE 1:**
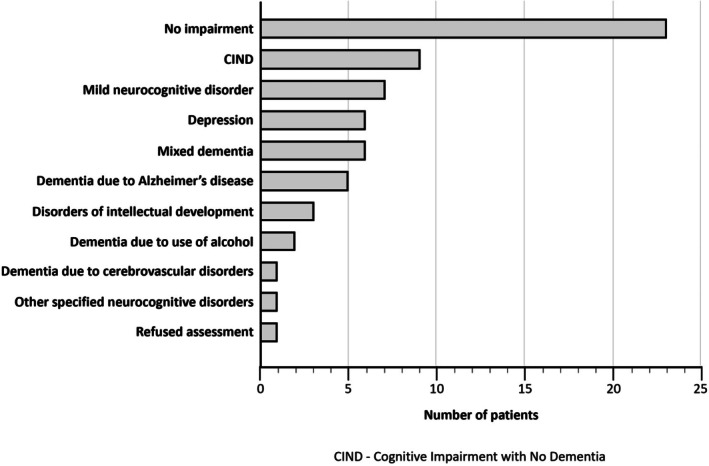
Diagnoses received by people attending the clinic.

Cognitive impairment was identified in 34 people (53%; 95% confidence interval [CI] 40.7%–65%), and a further six people (10%) were diagnosed with depression without cognitive impairment (Figure 1). Women were slightly more likely than men to have cognitive impairment (56% vs. 52%), although this was not statistically significant (*p* = 0.74). Among people with cognitive impairment, the most common diagnoses were cognitive impairment not dementia (CIND; 24%); mild neurocognitive disorder (21%); dementia due to Alzheimer's disease (15%); Alzheimer's disease dementia, mixed type (9%); and other mixed dementias (9%). One person who had previously been incorrectly diagnosed with dementia elsewhere was found to have normal cognition. Two‐thirds of people (71%; *n* = 24) with cognitive impairment were assessed with the MMSE. Their scores ranged from 9 to 29 (median 25.5; IQR 5), and nine people (38%) had a score less than 25. Twenty‐six of the 29 people (90%) without cognitive impairment were also assessed with the MMSE. Among this group, scores ranged from 25 to 30 (median 28; IQR 2).

Almost half of people seen at the clinic (44%; *n* = 28) were referred to additional services during their initial appointment. The most common referrals (which were not mutually exclusive) included: referral to disability services, home care package, or aged care assessment team (*n* = 10); imaging (six computed tomography [CT] scans and two magnetic resonance imaging [MRI] scans); and psychology or mental health services (*n* = 4). Less common referrals included: referral for antidepressant medication; exercise program; diabetes management; vascular surgeon for mixed vessel disease; social worker; nursing home care; physiotherapy; and neurology. People diagnosed with cognitive impairment received educational material about their diagnosis via post, and at least one follow‐up visit was usually scheduled to further discuss the diagnosis.

During the clinic's first 3 years of operation, two‐thirds of people (67%) attended only once, 17% attended twice, 11% attended three times, and three people (5%) attended four or more times. Among those who were followed up, one person with CIND was subsequently diagnosed with vascular dementia, one person with mild neurocognitive disorder subsequently had no impairment, and one person without initial cognitive impairment developed cognitive impairment after COVID‐19 and was subsequently diagnosed with mild neurocognitive disorder 4 months later. The remaining people were cognitively stable over the follow‐up period.

## 4 Discussion

This work demonstrates the success of a memory clinic located within an Aboriginal community‐controlled health service. The clinic was well‐attended, which stands in contrast with uptake at a nearby hospital‐based service. Sixty‐four Aboriginal people were seen in the first 3 years of the memory clinic's operation, compared with only five in the same period at Royal Perth Hospital, which is only 500 m away. Locating the memory clinic in a setting that is viewed as culturally safe by the Aboriginal community was likely to be one of the major enablers of attendance. Other enabling factors may include ACCHS assistance with transport when required, and patients having a trusted relationship with an AHP who gave clear information to support clinic access and was trained to conduct culturally appropriate cognitive screening. Aboriginal health practitioners are integral to the ACCHS model and to the memory clinic described in this article, acting as a cultural interface and facilitating trust and engagement of patients and their families [20]. As trusted health workers, AHPs were able to conduct home visits and collect detailed contextual information from family members.

Nonetheless, the memory clinic faced some challenges during its operation. There was a paucity of referrals at the time of initiation of the memory clinic which improved after staff and patients developed trust in the service. The clinic also experienced an interruption in support from the aged care assessment team (ACAT) system. A dementia specialist nurse initially attended the memory clinic to assess patients, but later withdrew due to funding constraints of the tertiary hospital. Patients' complex needs often resulted in prolonged attendances and many clients and their families required assistance navigating the aged care and National Disability Insurance Scheme (NDIS) pathways, including the need for medical assessments. Essential resources for the successful operation of a memory clinic within an ACCHS include funding for a geriatrician, an AHP (who can act as a Dementia Champion), a dementia specialize nurse and patient transport. Delivering a holistic service will require additional funding for allied health positions, such as a neuropsychologist, occupational therapist, speech therapist and social worker.

Most people living with dementia in Australia are women [1]. Accordingly, men are less likely to attend memory clinics, ranging from 25% to 47% of patients seen in previous Australian studies [21, 22, 23, 24, 25]. The proportion of male patients in this audit (47%) was at the high end of this range, which may indicate that Aboriginal men are at an increased risk of impaired cognition relative to their non‐Indigenous peers. This is supported by research in both urban‐ and remote‐living Aboriginal communities, which has found that Aboriginal men are at greater risk for dementia compared with women [26, 27]. Alternative explanations could include survivorship bias, more proactive assessment and referral of men to the memory clinic, or decreased uptake by women. However, slightly more female patients were found to have cognitive impairment than male patients, which remains consistent with the overall epidemiology of dementia in Australia [1].

A national survey of Australian memory clinics reported that Alzheimer's disease (38%), mild cognitive impairment (20%), mixed dementia (10%) and vascular dementia (9%) were the most common types of cognitive impairment identified, although diagnostic criteria for such diagnoses were not specified. No diagnosis was made for 4% of patients, and 7% had normal cognition [28]. A very different picture was evident in this audit, with 45% of patients found to have no cognitive impairment. Of those who were cognitively impaired, the most common diagnoses were CIND and mild neurocognitive disorder, which between them accounted for 47% of diagnoses. Dementia due to Alzheimer's disease and mixed dementia were less common. Dementia due to use of alcohol accounted for only 6% of diagnoses, although alcohol ingestion contributed to five of the mixed dementia diagnoses. The low prevalence of alcohol‐related dementia reflects the findings of research in the remote Kimberley region of Western Australia, which determined this dementia type is relatively rare in Aboriginal peoples [3], contrary to early (and potentially racially based) assumptions [29]. Dementia primarily due to cerebrovascular disease and chronic traumatic encephalopathy were relatively uncommon. Notable was the high proportion (33%) of patients who reported being members of the Stolen Generations. Out of all the states and territories of Australia, Western Australia has the highest proportion of Stolen Generation members, with 33% of Indigenous people aged 50 years and older having been removed from their family of origin [30]. Childhood trauma, such as that experienced by Stolen Generation survivors, may confer an increased risk of dementia [31]. Additionally, owing to past trauma, many survivors avoid clinical settings that resemble the environments they were removed to as children and fear re‐institutionalization. This may complicate care‐seeking and underscores the need for trauma‐informed care [32].

The majority of people attending the memory clinic were assessed with the MMSE. This tool is one of the most widely used methods to assess cognition worldwide, but may be inappropriate for some populations owing to literacy, language or cultural barriers [33]. Accordingly, the Kimberley Indigenous Cognitive Assessment (KICA) tool was developed and validated for older Aboriginal people living in regional and remote areas [16]. A modified version of the KICA tool for urban populations has similar predictive utility to the MMSE [34]. Assessment with the KICA tool typically takes 25–30 min. A short version of the KICA tool, the KICA Screen [35], can be completed in under 10 min and is comparable to the MMSE. We recommend that either the KICA Screen or the full KICA tool be used in similar memory clinics elsewhere.

This audit provides an important picture of the clinical profile and needs of older Aboriginal people attending an urban memory clinic. However, there are some limitations. Some older Aboriginal people may not access the health service in which the clinic was based for a variety of reasons, including caring responsibilities, a preference for other health services, and concerns regarding possible dementia diagnosis and resulting care. Concerns regarding institutionalization often impede assessment access, particularly in a generation that experienced forced removal from families [30]. As such, the sample may not necessarily be representative of older people with self‐, family‐, or clinician‐raised concerns about their cognition. Additionally, this clinical audit could not explore why patients preferred not to engage with mainstream services. This might reflect a need for tailored cultural safety training for staff working with older Aboriginal and Torres Strait Islander patients in mainstream services. Future research could potentially explore barriers and enablers to attendance.

## 5 Conclusions

In conclusion, a significant number of older Aboriginal people are not engaging with hospital‐based cognitive diagnostic services but will engage with a clinic located in, and supported by, an ACCHS. Aboriginal community‐controlled health services are an essential part of the health system and provide accessible, culturally safe care driven by community needs [14, 36]. This audit has demonstrated that a memory clinic located within such a service was well‐attended and fulfilled a need that was not being addressed by a hospital‐based clinic. Aboriginal community‐controlled health services may therefore be an ideal site for memory clinics, providing timely diagnosis, addressing potentially reversible causes of cognitive impairment, and improving health and aged care access for older Aboriginal people. Given the structural aging of the Aboriginal and Torres Strait Islander population [37], the need for memory clinics is likely to increase. The clinic described in this work could serve as a model that could be adopted by other Aboriginal health services. However, increased funding to support such initiatives is needed.

## Conflicts of Interest

Leon Flicker is a member of the Editorial Board of the Australasian Journal on Ageing. For all other authors, there are no conflicts of interest declared.

## Data Availability

The data that support the findings of this study are available on request from the corresponding author. The data are not publicly available due to privacy or ethical restrictions.
